# Medicinal Mushroom for Prevention of Disease of Modern Civilization

**DOI:** 10.1155/2015/812725

**Published:** 2015-04-19

**Authors:** Da-wei Qin, Zhengwei Gu, Jian-you Guo

**Affiliations:** ^1^School of Chemistry and Pharmaceutical Engineering, Qilu University of Technology, Jinan 250353, China; ^2^School of Pharmacy, Shandong University of Traditional Chinese Medicine, Jinan 250355, China; ^3^School of Pharmacy, Beijing University of Chinese Medicine 100029, China

Disease of modern civilization (DMC) such as type 2 diabetes and cardiovascular disease is caused by the pressure and tension, as well as nutritional imbalance, coupled with a lack of exercise, long-term cumulative a class of diseases. Medicinal mushrooms have diverse morphological, physiological, and ecological characteristics that support their diverse lifestyles. These specific interspecies interactions depend on the production of a wide range of bioactive substances. Medicinal mushrooms are well recognized for their medicinal properties and have been used in traditional medicine for millennia.

*Cordyceps sinensis* is an abundant resource in nature with various biological activities. Within the genus* Cordyceps*, over 400 species have been described so far, of which* Cordyceps sinensis*, also called “Winter Worm, Summer Grass,” is recognized as the most famous tonic herb in traditional Chinese medicine (TCM) for centuries. Many studies have shown that* Cordyceps sinensis* modulates immune responses, decreases plasma cholesterol levels, enhances hepatic function, regulates insulin sensitivity, and improves hypotensive and vasorelaxant activity. C. Han et al. review the chemical constituents and pharmacological actions of* Cordyceps sinensis*. Many bioactive components of* Cordyceps sinensis* have been extracted including nucleoside, polysaccharide, sterol, protein, amino acid, polypeptide, and others. In addition, these constituents' corresponding pharmacological actions were also shown in the study, such as anti-inflammatory, antioxidant, antitumour, antiapoptosis, and immunomodulatory. So we can use different effects of* C. sinensis* against different diseases and provide reference for the study of* Cordyceps sinensis* in the future. Y. Wang et al. investigated the effect of fermented mushroom of* Cordyceps sinensis* (CS) rich in selenium (Se-CS) on uterine cervical cancer in mice. Se-CS treatment (MCA and Se-0.4 group) showed a significant (*P* < 0.05–0.01) restoration in the level of the glutathione content, lipid peroxidation, glutathione peroxidase activity, glutathione reductase activity, catalase activity, Na+/K+-ATPase activity, and glutathione S transferase activity in MCA-induced tumor model (MCA-induced group). It may have helped the tissues recover from MCA injury. At the same time, Se-CS treatment (MCA and Se-0.4 group) results in significant reduction in the occurrence of cervical carcinomas (*P* < 0.05) compared with the MCA-induced group. This finding suggested that the concomitant use of Se and CS could be a potential therapeutic approach to improve the efficacy of therapy for uterine cervical cancer.

*H. erinaceum* is a temperate mushroom that has been domesticated and is commercially grown in China. The previous study demonstrated that this mushroom exhibited cytoprotection activity against ethanol-induced gastric ulcers in rats. Y. Xie et al. investigated the effects of* Hericium erinaceum* (HEM) on liver injury induced by acute alcohol administration in mice. HEM administration markedly (*P* < 0.05) decreased sera ALT, AST, and MDA levels. The hepatic histopathological observations showed that HEM had a relatively significant role in mice model, which had alcoholic liver damage. H. Yu-ling et al. investigated the effects of* Hericium erinaceum* (HEE) on alloxan induced diabetic neuropathic pain in laboratory rats. After 6 weeks of treatments, treatment with HEE 40 mg/kg in diabetic animals showed significant increase in pain threshold and paw withdrawal threshold and significant decrease in serum glucose and urine glucose. We also observed a significant increase in lactate dehydrogenase (LDH), lipid peroxidation (LPO), glutathione peroxidase (GPx) activity, glutathione reductase (GR) activity, catalase (CAT) activity, Na+K+ATPase activity, and glutathione S transferase (GST) activity along with significant decreased levels of glutathione (GSH) content in diabetic rats. The total antioxidant statuses (TAOS) in the HEE-treated groups were significantly lower than those in the alloxan-treated group.

*Inonotus obliquus* (PIO) is a mushroom habiting the cold latitudes of Europe and Asia, which was used as traditional Chinese medicine for a long history. In the last decade, several studies have reported biological activities of PIO such as anticancer, antioxidation, anti-inflammatory, and antihyperglycemic activities and enhancement of immunity. X. Yu et al. investigated the therapeutic effects of polysaccharides from* Inonotus obliquus* (PIO) on streptozotocin- (STZ-) induced diabetic symptoms and their potential mechanisms. The results show that administration of PIO can restore abnormal oxidative indices near normal levels. The STZ-damaged pancreatic *β*-cells of the rats were partly recovered gradually after the mice were administered with PIO 6 weeks later.

*Agaricus brasiliensis*, a mushroom of Brazilian origin, is widely used for nonprescript, medicinal purposes, both as an edible mushroom and in the form of extracts, which has been used as a health care product for the prevention of a wide range of illnesses including cancer, tumor, chronic hepatitis, diabetes, atherosclerosis, and hypercholesterolemia. A. P. de Santi-Rampazzo et al. investigated the effects of the supplementation with aqueous extract of* Agaricus blazei* Murrill (ABM) on biometric and blood parameters and quantitative morphology of the myenteric plexus and jejunal wall in aging Wistar rats. Supplementation with the ABM extract preserved the myenteric plexus in old animals, in which no differences were detected in the density and cell body profile of neurons and glial cells in the CA12 and CA23 groups, compared with C7 group. The supplementation with the aqueous extract of ABM efficiently maintained myenteric plexus homeostasis, which positively influenced the physiology and prevented the death of the neurons and glial cells.

*Grifola frondosa* (GF) is a Basidiomycete fungus belonging to the order Aphyllophorales and family Polyporaceae. It has recently attracted considerable attention for its various physiological activities. The extracts from the basidiomas of GF exerted a highly significant hepatoprotective effect by reducing the paracetamol-induced acute elevation of the AST and ALT levels. The agaricoglycerides (AG) are a new class of fungal secondary metabolites that constitute esters of chlorinated 4-hydroxy benzoic acid and glycerol. Agaricoglycerides showed strong activities against neurolysin, a protease involved in the regulation of dynorphin and neurotensin metabolism, and even exhibited anti-inflammatory and antinociceptive properties.

By compiling these papers, we hope to enrich our readers and researchers with respect to medicinal mushroom for disease of modern civilization.

*Da-wei Qin* *Da-wei Qin* *Zhengwei Gu* *Zhengwei Gu* *Jian-you Guo* *Jian-you Guo*

